# Shape and Subunit Organisation of the DNA Methyltransferase M.AhdI by Small-angle Neutron Scattering

**DOI:** 10.1016/j.jmb.2007.03.012

**Published:** 2007-05-25

**Authors:** P. Callow, A. Sukhodub, James E N Taylor, G.G. Kneale

**Affiliations:** 1 EPSAM and ISTM Research Institutes, Keele University, Staffordshire ST5 5BG, UK; 2 ILL-EMBL Deuteration Laboratory, Partnership for Structural Biology, Institut Laue Langevin, 38042 Grenoble Cedex 9, Grenoble, France; 3 Biophysics Laboratories, Institute of Biomedical and Biomolecular Sciences, University of Portsmouth, PO1 2DT, UK

**Keywords:** restriction-modification, type I DNA methyltransferase, contrast variation, perdeuteration, multi-subunit enzymes

## Abstract

Type I restriction-modification (R-M) systems encode multisubunit/multidomain enzymes. Two genes (M and S) are required to form the methyltransferase (MTase) that methylates a specific base within the recognition sequence and protects DNA from cleavage by the endonuclease. The DNA methyltransferase M.AhdI is a 170 kDa tetramer with the stoichiometry M_2_S_2_ and has properties typical of a type I MTase. The M.AhdI enzyme has been prepared with deuterated S subunits, to allow contrast variation using small-angle neutron scattering (SANS) methods. The SANS data were collected in a number of ^1^H:^2^H solvent contrasts to allow matching of one or other of the subunits in the multisubunit enzyme. The radius of gyration (*R*_g_) and maximum dimensions (*D*_max_) of the M subunits *in situ* in the multisubunit enzyme (50 Å and 190 Å, respectively) are close of those of the entire MTase (51 Å and 190 Å). In contrast, the S subunits *in situ* have experimentally determined values of *R*_g_ = 35 Å and *D*_max_ = 110 Å, indicating their more central location in the enzyme. *Ab initio* reconstruction methods yield a low-resolution structural model of the shape and subunit organization of M.AhdI, in which the Z-shaped structure of the S subunit dimer can be discerned. In contrast, the M subunits form a much more elongated and extended structure. The core of the MTase comprises the two S subunits and the globular regions of the two M subunits, with the extended portion of the M subunits most probably forming highly mobile regions at the outer extremities, which collapse around the DNA when the MTase binds.

## Introduction

Type I restriction-modification (R-M) systems encode multisubunit/multidomain enzymes that recognize an asymmetric bipartite DNA sequence.^1^ They comprise three genes, one for each of the subunits (S, M and R) that are responsible for specificity, methylation and restriction, respectively. Two genes (M and S) are required to form the trimeric methyltransferase (MTase), M_2_S, that methylates a specific base within the recognition sequence and protects the DNA from cleavage by the endonuclease.2., 3. Sequence specificity is conferred by the two target recognition domains (TRDs) of the S subunit, each binding a half-site within the DNA recognition sequence. The corresponding endonuclease is a pentameric enzyme, formed from the MTase by the addition of two R subunits to form a complex of stoichiometry R_2_M_2_S with a typical mass of around 400 kDa.4., 5.

The related MTase, M.AhdI, from *Aeromonas hydrophila* has an organization similar to that of type I MTases but differs in having identical TRDs, which in this case are on separate subunits, each corresponding roughly to half of a classical S subunit. The enzyme has the stoichiometry M_2_S_2_ and recognizes and methylates the symmetrical DNA sequence, GACN_5_GTC.^6^ The S and M subunits of M.AhdI are 25 kDa and 60 kDa, respectively, and have been well characterized both biochemically and biophysically. M.AhdI can be reconstituted from separately expressed M and S subunits, and the reconstituted enzyme has been shown to have DNA methylation activity *in vitro*.^6^ The multisubunit complex has been fully characterized by analytical ultracentrifugation and dynamic light-scattering,^6^ having a sedimentation coeficient of 7.8 S, a hydrodynamic radius of 5 nm, and a molecular mass of 170 kDa, similar in size to M.EcoKI and M.EcoR124I.

There is no high-resolution structure available for any intact type I MTase, although the structures of the putative S subunits of *Methanococcus jannaschii*^7^ and *Mycoplasma genitalium*^8^ have been determined recently by X-ray crystallography. However, in neither case was the protein shown to be a component of an MTase. Indeed in the case of the putative S subunit from *M. genitalium,* there seems to be no corresponding M subunit encoded in the genome and thus the function of this protein is unclear; it may have some other DNA-binding role, unrelated to R-M activity. Nevertheless, the overall features of these structures are similar, and are likely to apply to the S subunits of other, well-characterized, MTases. In both structures, the two TRDs form globular domains linked by two antiparallel α helices, corresponding to the two conserved domains of the protein. Both structures have a circular topology, as predicted on theoretical grounds, with the N and C termini of the polypeptide in close proximity.^9^ The two TRDs of the S subunit are in an orientation appropriate to fit into the major groove of DNA, as anticipated.

The X-ray crystal structure of an M subunit is available in the Protein Structure Databank (PDB ID 2AR0) but the structure has not been published. In the crystal, the M subunits form a symmetrical dimer, although it is not known whether the protein is dimeric in solution. Significantly, substantial parts of the structure are unresolved in the electron density map, suggesting the presence of highly mobile regions. The M subunits of both M.EcoKI and M.EcoR124I are susceptible to limited proteolysis, an indication of the presence of flexible and/or unstructured regions,10., 11. and this may be a general feature of such subunits.

A number of quite different models have been proposed for type I MTases, based on partial homology to various subunits or domains of known structures.7., 8., 12., 13. In the absence of any experimental structure for an intact type I MTase, small-angle scattering can be employed to investigate the overall shape of the enzyme. Small-angle X-ray scattering (SAXS) experiments on M.EcoR124I revealed an elongated structure with an overall radius of gyration (*R*_g_) of 56 Å and a maximum dimension of 180 Å.^14^

A limitation of SAXS is the difficulty in determining the locations of individual subunits, even if the overall shape can be determined. However, with small-angle neutron scattering (SANS), individual subunits can be perdeuterated to permit the use of contrast matching.^15^ Thus, for example, by reconstitution of the MTase with hydrogenated M subunits and deuterated S subunits, and measuring scattering curves in 40% ^2^H_2_O, the M subunits are “matched out” and the structure and location of the S subunits within the MTase can be analysed. Likewise, in 100% ^2^H_2_O, one essentially sees the structure of the M-subunits within the selectively deuterated enzyme.

Unlike R-M systems such as EcoR124I, both the M and S subunits of M.AhdI are sufficiently soluble to allow reconstitution of the enzyme from separately expressed subunits, which can be differentially deuterated before reconstitution. In order to determine the arrangement of the subunits of the methyltransferase, we have prepared M.AhdI in two states for SANS experiments: the first as a fully hydrogenated enzyme, the second with the M subunit hydrogenated and the S subunits perdeuterated. By varying the ^1^H:^2^H content of the solvent, the selectively labeled subunits can be contrast-matched and scattering data collected for the individual subunits *in situ* in the MTase complex. From such experiments, we have determined the low-resolution shape of the M and S subunits in the complex and the location of these subunits in the MTase.

## Results

### SANS analysis

**Figure 1:**
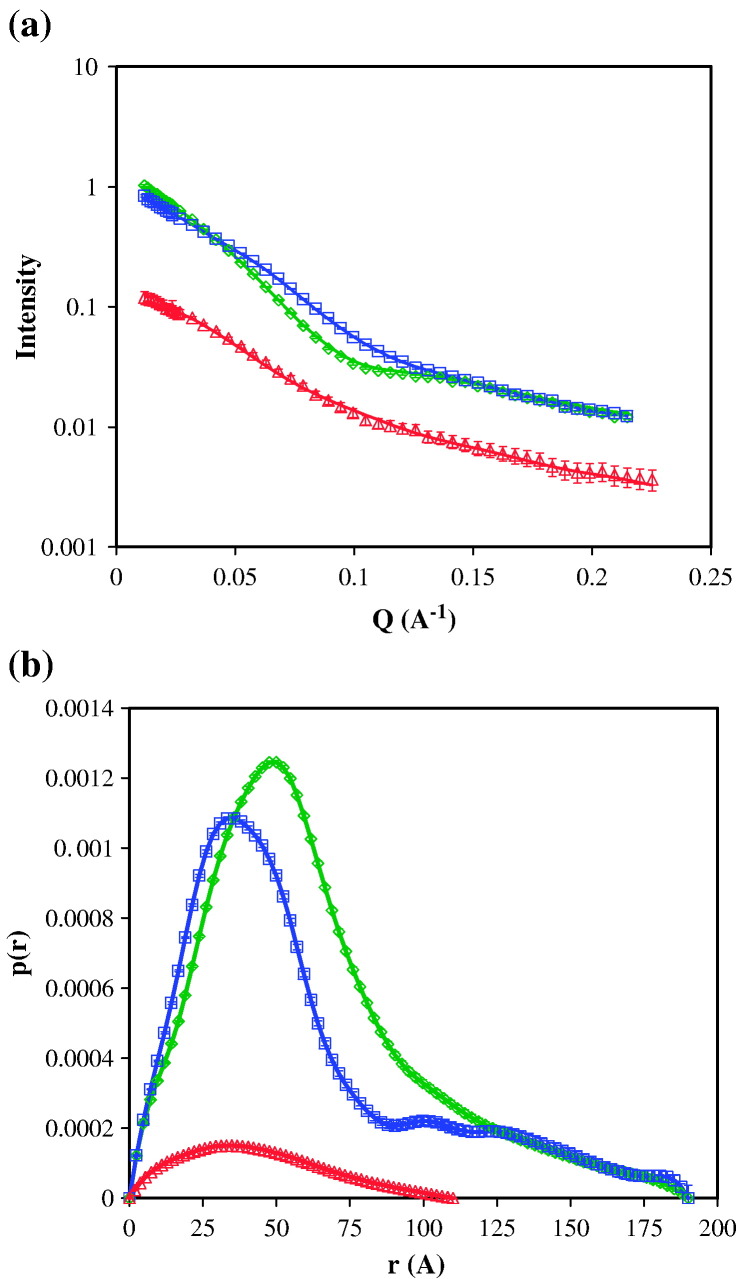
(a) SANS data for hydrogenated M.AhdI in 100% ^2^H_2_O (green), and for M.AhdI with the S subunit deuterated/M subunit hydrogenated in 100% ^2^H_2_O (blue) and in 40% ^2^H_2_O (red). The continuous lines are the model fits resulting from *ab initio* shape determination using DAMMIN.^15^ (b) Distance distribution functions calculated for the SANS data using GNOM.^20^

Firstly, data were collected for the hydrogenated M.AhdI enzyme in 100% ^2^H_2_O (Figure 1(a)). The scattering data can be transformed into a distance distribution function, *P*(r), which shows the distribution of all inter-atomic vectors in the molecule (Figure 1(b)). This allows us to determine the *R*_g_ and the longest dimension (*D*_max_) of the entire complex. For M.AhdI, the *R*_g_ was found to be 51( ± 1) Å and the *D*_max_ was 190 Å; these values are of a magnitude similar to those determined for the EcoR124I MTase by SAXS (56 Å and 180 Å, respectively).

Scattering data were collected for an M.AhdI sample in which the S subunits were perdeuterated and the M subunits were hydrogenated (Figure 1(a)). Measurements were taken at two solvent contrasts: 40% ^2^H_2_O, where the hydrogenated M subunits of the complex are contrast-matched and therefore do not contribute to the scattering pattern, and 100% ^2^H_2_O, where the contribution of the deuterated S subunits to the scattering pattern is minimal. The corresponding distance distribution curves, *P*(r), are shown in Figure 1(b). Table 1 shows the *R*_g_ and *D*_max_ values calculated for the native enzyme and the selectively perdeuterated M.AhdI enzyme in 40% ^2^H_2_O and 100% ^2^H_2_O, the latter corresponding to values for the S subunits and the M subunits, respectively, *in situ* in the MTase.

**Table 1:** *R*_g_ and *D*_max_ parameters from SANS of M.AhdI

	*R*_g_ (Å)	*D*_max_ (Å)
MTase^a^	51 ± 1	190 ± 5
M subunits^b^	50 ± 1	190 ± 5
S subunits^c^	35 ± 1	110 ± 5

a M.AhdI in 100% ^2^H_2_O.

b M.AhdI with deuterated S subunits in 100% ^2^H_2_O.

c M.AhdI with deuterated S subunits in 40% ^2^H_2_O.

For the selectively perdeuterated M.AhdI in 100% ^2^H_2_O, the S subunits are effectively contrast-matched and so only the M subunits contribute substantially to the scattering. The similarity in both *R*_g_ and *D*_max_ for the M subunits and the intact MTase (Table 1) implies that only the M subunits are contributing to the largest inter-atomic distances within the M.AhdI complex. When the M subunits are contrast matched in 40% ^2^H_2_O, only scattering from the S subunits is observed. It is clear that the S subunits have considerably smaller *D*_max_ (110 Å) and *R*_g_ (35 Å) values than those of the M subunits (190 Å and 51 Å, respectively). Thus, the M subunits extend towards the outside of the complex, while the S subunits are located more centrally.

For comparison, *R*_g_ and *D*_max_ of the related restriction-modification subunits have been calculated from the available crystal structures of the *M. jannaschii* S subunit (PDB code 1YF2), and the EcoKI M subunit dimer (PDB code 2AR0), (see Table 2). In each case, the *R*_g_ was calculated for the crystal structure for a hydrogenated protein in 100% deuterated buffer, assuming 10% of the protein hydrogen atoms were non-exchangeable.

**Table 2:** Calculated values of *R*_g_ and *D*_max_ from X-ray crystal structures

	*R*_g_ (Å)	*D*_max_ (Å)
M subunit, EcoKI (dimer)	40	151
S subunit, MjaI	29	88

The values were calculated using the program CRYSON.^22^

If one compares the values determined by SANS for the selectively deuterated M.AhdI (where the *R*_g_ and *D*_max_ values for the M and S subunits are determined *in situ*) with the values calculated from the crystal structures of the equivalent subunits, we observe that in both instances the values of *R*_g_ and *D*_max_ are larger for the SANS-derived structures. The increases in *R*_g_ are much larger than any possible effects to due hydration, which are generally minimal for SANS.^22^ However, they could reflect a difference in structure between the subunits of M.AhdI and those of the *M. jannaschii* and/or EcoKI enzymes, discrepancies between solution and crystal structures or structural differences between the free subunits and the subunits *in situ* in the MTase (see Discussion).

The latter possibility could, in principle, be investigated by solution scattering experiments on the isolated subunits. However, the M subunit of AhdI aggregates at high concentrations of protein and consequently is unsuitable for small-angle scattering studies in free solution, although the AhdI S subunit is much more soluble. We therefore carried out SAXS on the isolated AhdI S subunit dimer (data not shown). Analysis of the SAXS data gave a value of *R*_g_ = 35( ± 0.5) Å, in excellent agreement with the value obtained for the S subunit dimer *in situ* by SANS. Thus the discrepancy in *R*_g_ between the latter and the value of 29 Å for the *M. jannaschii* S subunit arises most probably from the larger size of the AhdI S subunit dimer (51 kDa compared to 48 kDa), rather than any gross conformational change when forming the MTase.

### *Ab initio* shape determination

*Ab initio* shape determination has been performed for the data obtained under different contrast conditions using DAMMIN,^16^ a program that employs simulated annealing to restore the solution structure from solution scattering curves. The resulting model consists of dummy atoms defining the shape of the macromolecule at an appropriate resolution. For each data set, the modeling program was run 20 times and the resulting shapes averaged and filtered to give the final shape.

**Figure 2:**
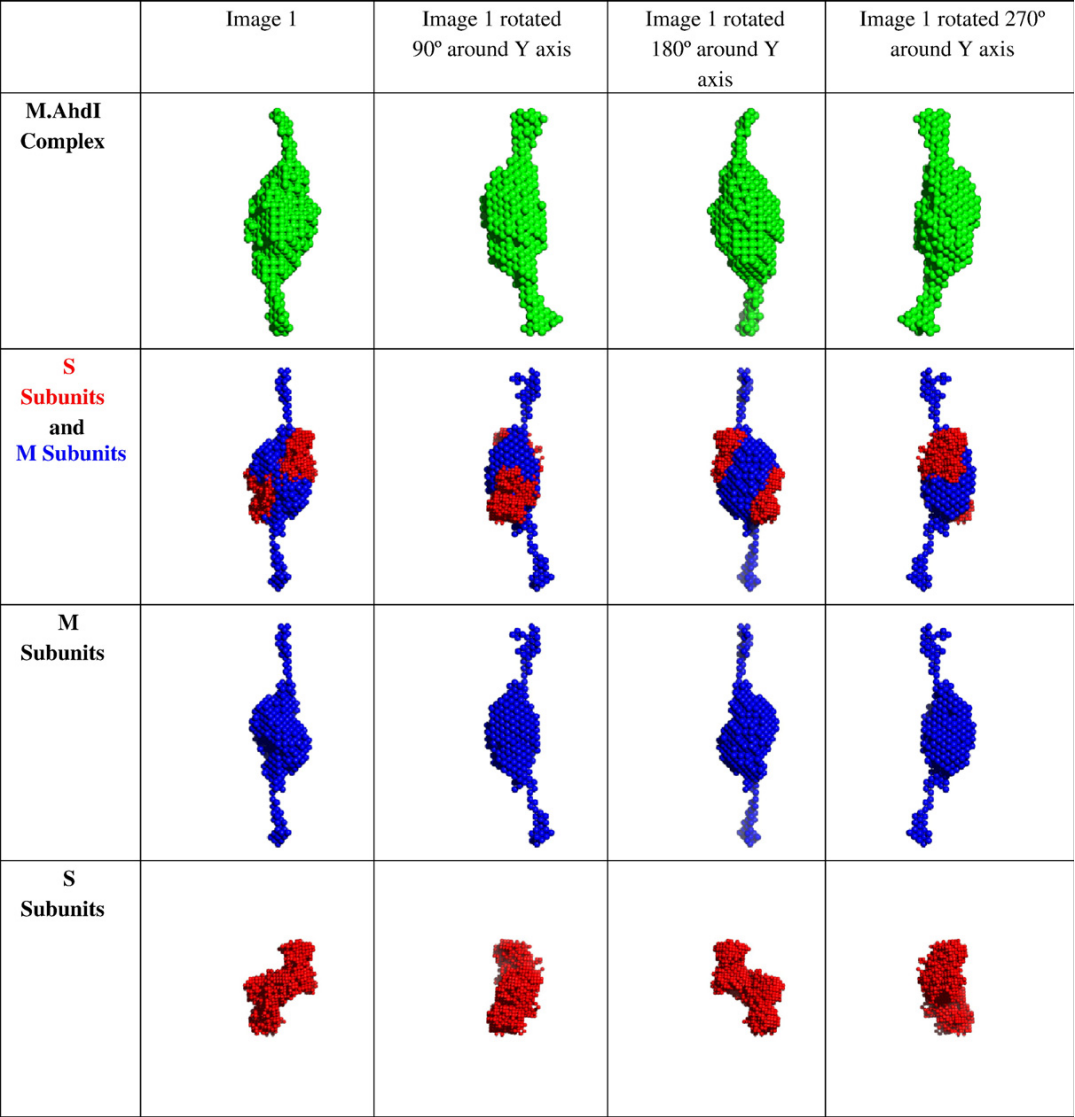
Low-resolution *ab initio* models for M.AhdI and for its subunits *in situ* derived from contrast variation SANS experiments. Views for each structure correspond to 90° rotations about the vertical axis.

The *ab initio* shape determined for the M.AhdI complex is markedly elongated, with a central core that is more globular (Figure 2). The *ab initio* model for the selectively perdeuterated enzyme in 100% ^2^H_2_O (i.e. when the S subunits are contrast-matched) indicates the shape of the M subunits within the complex. When this shape is aligned with the shape for the entire complex, it is possible to assign the M subunits to various regions of the complex. As was inferred from the distance distribution functions (Figure 1(b)), the M subunits are located predominantly along the longest axis of the shape determined for the M.AhdI complex. The shape determined for the S subunits (i.e. when the M subunits are contrast-matched in the selectively deuterated complex) fits a region of the envelope determined for the whole complex that is not occupied by the M subunit (Figure 2).

Rigid-body modeling provides an alternative approach to *ab initio* modeling. This, however, requires that the structures (or sub-structures) of the isolated subunits are good models for those of the multisubunit complex. Attempts were made to fit the SANS data for M.AhdI by rigid-body modeling, based on the available crystal structures of the homologues of the S and M subunits. These attempts included allowing the position and orientations of each M subunit to vary independently, as well as keeping the crystallographic dimer as one unit. We also allowed the inner and outer domains of the M subunit to move independently. However, in none of these cases was the fit to the data satisfactory, and the resulting structures did not look sensible. We conclude that the available structures are not appropriate for rigid-body modeling of M.AhdI. The reported crystal structures for the M dimer of EcoKI and the S subunit of MjaI may not be sufficiently good models for the solution structure of M.AhdI, since there is only weak sequence homology (as discussed below). Moreover, significant parts of the structure of the M subunit of M.EcoKI are missing in the reported crystal structure, and there is significant conformational flexibility.

## Discussion

**Figure 3:**
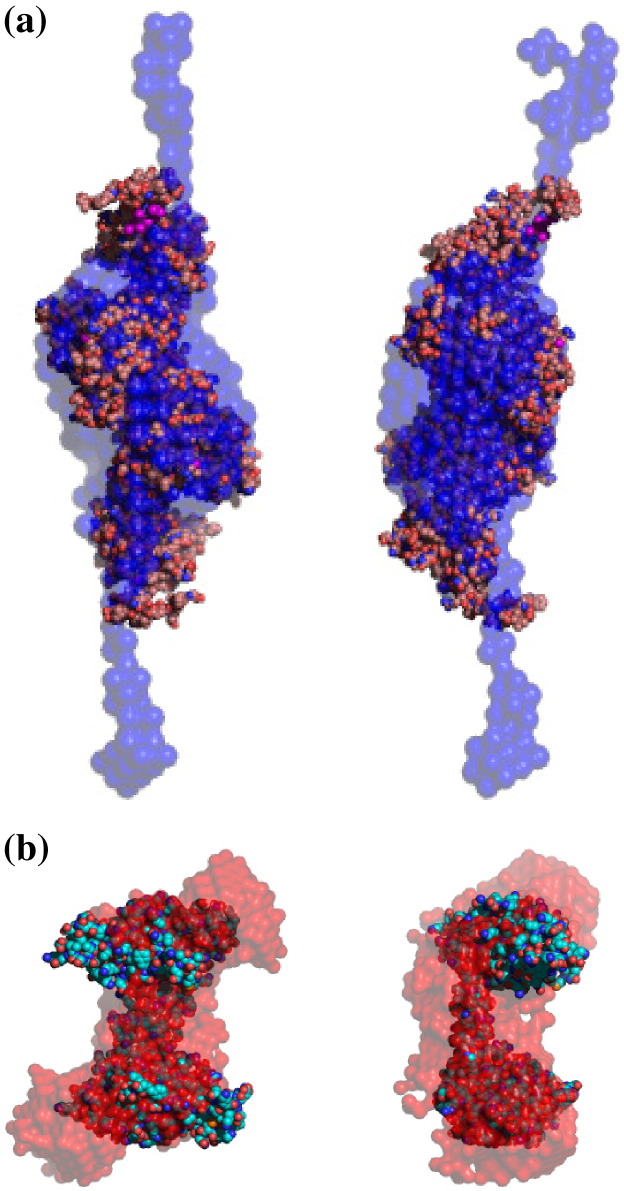
Comparison of *ab initio* models for M.AhdI subunits *in situ* with structures of related subunits determined by X-ray crystallography. (a) Dimer of the M subunits of AhdI (pale blue) and EcoKI (dark blue where structures overlap). (b) Dimer of the S subunits of AhdI (pale red) and the monomeric S subunit of *M. jannaschii* (dark red where structures overlap).

The structure we have determined for M.AhdI represents the first experimental structure of any type I MTase, albeit at low resolution. From the overall shape of the multi-subunit enzyme, the location of the subunits (and their domains) cannot be determined, since they are in intimate contact. However, by employing specific deuteration/contrast variation techniques, the location of the M and S subunits becomes apparent. The dimer of AhdI S subunits (each equivalent to half a classical S subunit) has the Z-shaped structure that has been observed in other (putative) S subunits at high resolution by X-ray crystallography. The M subunit is much more extended, with a globular core in contact with the S subunits and an extended outer region that is responsible for the high *D*_max_. Figure 3 shows the structures of the M and S subunits within the multisubunit M.AhdI as determined by SANS, superimposed on the structures of their homologues.

Comparison of the shape of the M.AhdI M subunit dimer determined by SANS with the crystal structure of the equivalent EcoKI M-dimer shows that the outer extended regions of the AhdI structure are not present in the EcoKI structure (Figure 3(a)). Indeed, this is evident from the 40 Å difference between the *D*_max_ for the two (see Table 1, Table 2). It should be noted that the crystal structure of the M subunits of EcoKI shows significant disorder. In this structure, residues 150–474 are located in the central domain and residues 1–117 and 482–527 appear to make up the outer domain. There is substantial missing density in the map, notably in the interdomain region (residues 118–149), suggesting that the outer domains are extremely flexible, and might therefore be subject to considerable crystal packing effects. Thus, structural differences between the X-ray crystal structure of EcoKI M subunits and the M subunits observed *in situ* in the solution structure of M.AhdI are not unexpected. Unstructured and highly flexible regions may be a common feature of the M subunits of type I MTases, and could play a functional role. Indeed, it has been proposed that the large (∼60 Å) reduction in dimensions of M.EcoR124I observed by SAXS may be due to the outer regions of the M subunits collapsing in to surround the DNA.^14^

**Figure 4:**
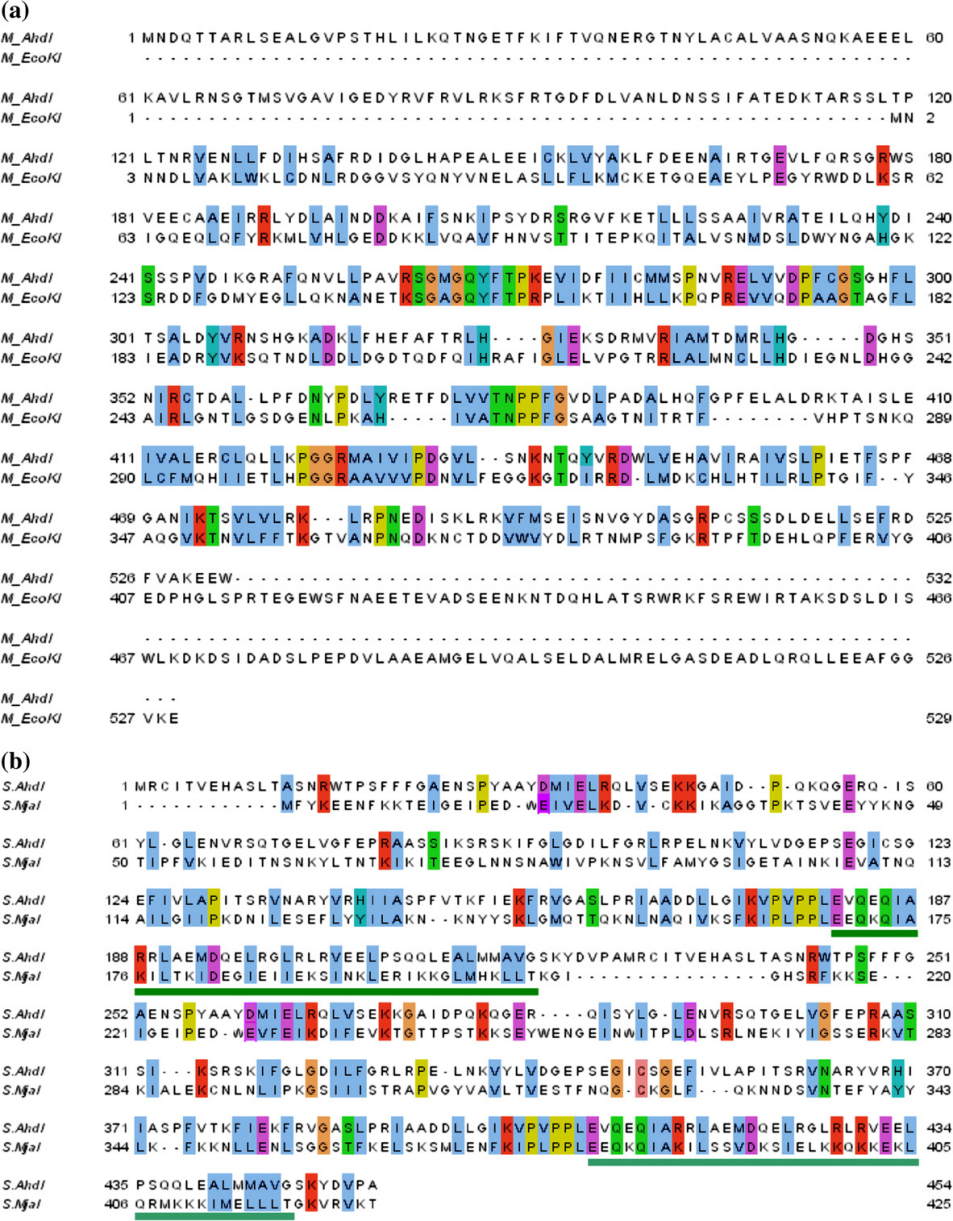
Sequence alignments of the M subunits of AhdI and EcoKI, and the S subunits of AhdI and *M. jannaschii*. (a) Alignment of the M subunits of AhdI and EcoKI. (b) Alignment of a dimer of AhdI S subunits and a monomer of the S subunit of *M. jannaschii*. The coiled-coil regions corresponding to the central conserved region (CCR, dark green) and distal conserved region (DCR, light green) are indicated, based on the crystal structure of the *M. jannaschii* S subunit. Amino acid sequences in both cases were aligned using Blast2seq,^25^ and identical or similar amino acid residues are colored according to the ClustalX^26^ color scheme using Jalview.^27^

Both AhdI and EcoK M subunits are very similar in size (532 and 529 amino acid residues, respectively) but comparison of their sequences shows that they have only weak overall homology (Figure 4(a)). There are nevertheless four regions of distinct homology (each ∼10 residues) in the central region of the two proteins, sufficient to align the sequences, and over AhdI residues 261–503, there is 30% identity and 46% similarity. On this alignment, there is an additional sequence of 118 residues at the N terminus of the AhdI sequence and likewise a stretch of 117 residues at the C terminus of the EcoKI sequence. Although these two regions show no clear sequence homology, they could be structurally homologous. Indeed, the two M subunits could be related by circular permutation, with the N and C termini of the polypeptide in close proximity (analogous to the organization of domains in type I S subunits,^9^ but in this case without the symmetry arising from the direct repeat). The fact that the outer domain of the EcoKI M subunit appears to be made up of regions from the N and C-terminal sequences of the polypeptide would support this proposition.

From the comparison shown in Figure 3(b), it can be seen that the overall shape of the S subunit dimer in the AhdI MTase resembles that of the recently determined crystal structure of the presumed S subunit of *M. jannaschii*,^7^ in which the two domains corresponding to the TRDs are linked by a spacer region. The two S subunits of AhdI correspond to the single S subunit of MjaI in the crystal structure, consistent with the two conserved domains forming a coiled-coiled structure similar to that of classical type I S subunits, but in this case on separate polypeptide chains. Nevertheless, the shape of the AhdI S subunit dimer as determined by SANS is larger than the MjaI S subunit seen in the crystal structure, as was indicated also by an increase in *R*_g_. As discussed above, the larger shape of the SANS model for the S subunit dimer of AhdI is not due to changes in structure on forming the MTase, since SAXS experiments on the isolated dimer give essentially the same *R*_g_. It may reflect structural differences between the two species and/or the effects of averaging structures in solution.

Comparison of the sequences of the AhdI and *M. jannaschii* S subunits (Figure 4(b)) reveals fairly weak homology over most of the sequence (over the region of AhdI with the highest level of homology, residues 128–434, there is 20% sequence identity and 39% similarity). In the alignment shown, two copies of the AhdI sequence have been linked together as a covalent dimer, to simulate the repeated sequences of regular type I S subunits (this sequence is 454 residues compared to 422 residues in the MjaI subunit). There are two repeated proline-rich sequences, highly conserved between all type I S subunits, that show particularly strong homology between the AhdI and MjaI sequences, centered on the motif PL(V)PPLE. In the crystal structure of the MjaI subunit, these sequences correspond to the hinge region that connects the C-terminal end of each TRD with the N-terminal end of the adjacent coiled-coil spacer. There is also a region of partial homology in the repeated sequence that corresponds to the link from the N-terminal region of each TRD to the C-terminal end of the coiled-coil spacer. In the crystal structure of S.MjaI, these two regions (denoted β1′^+^ and β1′^−^) at the N and C terminii of each TRD interact to form anti-parallel β-ribbons at the entrance to the TRD. On the alignment shown, all seven of the hydrophobic residues in each TRD^7^ that interact with the corresponding hinge regions (PLPPL) in the corresponding TRD are conserved between S.AhdI and S.MjaI, as also are five of the six apolar residues making up the hydrophobic pocket of each TRD that was suggested as a possible interaction site for the M subunits.^7^

On this alignment of the two sequences (Figure 4(b)), 12 of the additional 16 residues (per AhdI subunit) that account for the larger size of this subunit are found at the N terminus, corresponding to the start of each TRD. Some of these residues could participate in (and thus extend) the coiled-coil spacer, but it is likely that the bulk of these residues would form additional structure in this region that may or may not interact with the remainder of the TRD. Indeed, a comparison of the structures of the S subunits of AhdI and MjaI shows that the shape of each TRD in S.AhdI is extended at either end of the coiled-coil spacer (Figure 3(b)). The additional residues could account for at least some of the increase in the *R*_g_ of the AhdI dimer compared to the S subunit of *M. jannaschii*.

In summary, we have elucidated the first low-resolution structure of a type I MTase, making use of specific subunit deuteration and contrast variation to reveal the location of individual subunits. The overall shape of the enzyme in solution shows a compact structure, approximately 100 Å × 60 Å × 50 Å, comprising the two S subunits and the core domains of the two M subunits. However, the outer regions of the M subunits extend the longest dimension of the MTase to 190 Å. It is proposed that these extended regions of the M subunits in type I MTases are flexible and collapse around the DNA to form a more globular structure in the MTase-DNA complex, consistent with the large conformational change deduced from SAXS for M.EcoR124I.^14^ It would also offer an explanation for the large DNAse I footprint,^17^ indicating that ∼23 bp (80 Å) of the DNA are almost completely enclosed in the DNA–protein complex.

## Materials and Methods

The plasmids encoding the M and S subunits of the M.AhdI complex were transformed into BL21(DE3) cells, from which they can be over-expressed. The bacteria were then grown on Enfors minimal medium using an Infors fermentation system at 30 °C to an *A*_600_ of ∼15. Glycerol was used as the carbon source; for the hydrogenated protein h8-glycerol was used and d8-glycerol was used for the expression of the perdeuterated S subunit. The H_2_O in the medium was replaced with ^2^H_2_O for the perdeuteration of the protein. Purification of the AhdI MTase was performed by combining cell pellets from M and S-expressing cells, and purifying the intact enzyme from cell lysates as described by Marks *et al*.^18^ The monodispersity of samples was checked routinely by dynamic light-scattering, to confirm the presence of a single species with a hydrodynamic radius of ∼5 nm in agreement with previous measurements.^6^

Data were collected using the D22 diffractometer at the ILL (with two detector distances covering a *Q* range of 0.01–0.25 Å^−1^). Data reduction was performed using the GRAS_ans_P software (Dewhurst, 2006)†. Using the Guinier approximation to determine the *I*_o_ value for each sample in each ^2^H_2_O:H_2_O solvent contrast, we established the contrast match points for the hydrogenated and deuterated protein within the M.AhdI complex.^19^ For the hydrogenated enzyme, the contrast match point was found to be 41% ^2^H_2_O and for the partially deuterated enzyme, 89% ^2^H_2_O, confirming the successful incorporation of the deuterated subunits.

Modeling of the SANS data was performed using the ATSAS software package developed by Svergun *et al.*^22^ Distance distribution functions, *p*(r), were calculated using GNOM.^20^ Having calculated *R*_g_ directly from the scattering curves using the Guinier approximation, multiple *p*(r) functions were calculated using the program GNOM, with *D*_max_ varying from 80 Å to 220 Å for each of the data sets. Scattering curves were then generated by back transformation of each of these *p*(r) functions and compared to the experimental data. The value of *D*_max_ finally chosen was the value that gives an *R*_g_ that matches most closely the experimental R_g_ determined from Guinier plots.

*Ab initio* shape determination was performed using DAMMIN,^15^ which uses simulated annealing to calculate single-phase dummy atom models. DAMMIN was run in expert mode and the default values were used except where noted otherwise. A prolate ellipsoid was defined with semi-axes of 95 Å and 55 Å composed of 3842 dummy atoms, each with radius of 3.8 Å. *P*2 symmetry was imposed on the ellipsoid and the simulated annealing procedure was run with the schedule factor (which determines the rate of convergence of the iteration) set to 0.9. A penalty weight of 4 × 10^−3^ for the looseness and disconnectivity parameters was applied to the resulting models, and for the peripheral penalty weight, a value of 0.3 was applied. Initially, the shape of the entire complex was modeled using the data collected for hydrogenated M.AhdI in 100% deuterated buffer. Models with *R*_f_, Looseness and disconnectivity values of greater than 0.01, 0.10 and 0.00, respectively were discarded. The resulting models were used as the starting template to model the M and S subunits, using the data collected for M.AhdI, where the S subunit was selectively deuterated and data collected in 100% and 40% deuterated buffer, respectively. For each of these data sets, the data were modeled to a *Q* value of 0.22 Å^−1^ and the 2-fold symmetry axis maintained.

Each data set was modeled 20 times and the resulting shapes were aligned, averaged and filtered using the DAMAVER package of programs.^21^ The volume of the resulting model is inevitably larger than the actual molecular volume, due to the averaging process and to the low resolution of the model. In practice, the cut-off volume for the resulting shape after filtering is varied until the calculated *R*_g_ (using the program CRYSON^22^) of the shape corresponds to that determined experimentally.

Once *ab initio* shapes had been determined for each of the subunits of M.AhdI and for the complex itself the first stage of the alignment was performed computationally. The M subunits were aligned to the MTase using the program SUPCOMB20^23^ and checked by manual inspection. Once satisfied that the alignment was correct, dummy atoms that coincided with the shape for the MTase and the aligned shape for the M subunits were removed from the model for the MTase complex. The remaining dummy atoms should correspond to density resulting from the S subunits, so at this stage the shape determined for the S subunits was aligned computationally with the remaining dummy atoms from the MTase. As a final check, *R*_g_ was calculated for the shape defined by the two aligned shapes representing the S and M subunits, and was in agreement with that seen for the M.AhdI complex.

The alignment of the available crystal structures with the *ab initio* models of the subunits was performed both manually and computationally using SUPCOMB20.^23^ All visualisations of PDB files were performed using PyMol‡.

Rigid-body refinement was performed using the program MASSHA.^24^ Models were prepared for the MTase using the available high-resolution structures of both M and S subunits with and without the imposition of 2-fold symmetry and compared to the SANS scattering curves. Additionally, the M subunit dimer was separated into separate monomers and allowed to rotate and translate independently. Finally, the M subunit monomers were separated into major and minor domains, and allowed to fit independently.

## Footnotes

† http://www.ill.fr/lss/grasp/grasp_main.html

‡ http://www.pymol.org
